# ﻿Taxonomic notes on the genus *Phrynarachne* from China (Araneae, Thomisidae)

**DOI:** 10.3897/zookeys.1085.77966

**Published:** 2022-02-04

**Authors:** Yejie Lin, Long Yu, Peter Koomen, Xunyou Yan, Shuqiang Li

**Affiliations:** 1 Hebei Key Laboratory of Animal Diversity, College of Life Science, Langfang Normal University, Langfang 065000, China Langfang Normal University Langfang China; 2 State Key Laboratory of Biocatalysis and Enzyme Engineering of China & Centre for Behavioural Ecology & Evolution, School of Life Sciences, Hubei University, Wuhan 430062, China Hubei University Wuhan China; 3 Natuurmuseum Fryslân, Schoenmakersperk 2, Leeuwarden, 8911 EM, The Netherlands Natuurmuseum Fryslân Leeuwarden Netherlands; 4 Institute of Zoology, Chinese Academy of Sciences, Beijing 100101, China Institute of Zoology, Chinese Academy of sciences Beijing China

**Keywords:** Diagnosis, new species, nomen dubium, type specimens

## Abstract

Four new species of the genus *Phrynarachne* Thorell, 1869 from China are described: *P.dreepy* Lin & S. Li, **sp. nov.** (♂♀), *P.xuxiake* Lin & S. Li, **sp. nov.** (♀), *P.yunhui* Lin & S. Li, **sp. nov.** (♀), and *P.zhengzhongi* Lin & S. Li, **sp. nov.** (♀). The unknown sexes of *P.brevis* Tang & S. Li, 2010 (♂), *P.huangshanensis*Li et al., 1985 (♀), *P.lancea* Tang & S. Li, 2010 (♂), and *P.mammillata* Song, 1990 (♀) are described for the first time. *Phrynarachnesinensis* Peng, Yin & Kim is treated as a nomen dubium.

## ﻿Introduction

The spider genus *Phrynarachne* Thorell, 1869 currently includes 32 species and subspecies distributed in southern Asia, the Australian region, and sub-Saharan Africa. Only five species are described by both sexes, and 10 species have been studied after their original description. Efforts have been made to find *Phrynarachne* types preserved in well-known European museums, but these endeavors have failed.

Seven *Phrynarachne* species were known from China before the current study; only two species, i.e., *P.ceylonica* (O. Pickard-Cambridge, 1884) and *P.katoi* Chikuni, 1955, are described by both sexes. All endemic Chinese *Phrynarachne* species are only described by few single-sex specimens, and the species in the surrounding areas of China, except Japan, are not well revised and most of them have only initial descriptions (Li et al. 2021; WSC 2021; Yao et al. 2021).

Here, we describe four new and six known *Phrynarachne* species from China. Due to the lost holotype and unknown locality in the original description, we treat *P.sinensis* Peng et al. as *nomen dubium*.

## ﻿Materials and methods

All specimens were preserved in 80% ethanol. Epigynes were cleared in trypsin enzyme solution to dissolve non-chitinous tissues. Specimens were examined under a LEICA M205C stereomicroscope. Photomicroscopy images were taken with an Olympus C7070 zoom digital camera (7.1 megapixels). Laboratory habitus photographs were taken with a Sony A7RIV digital camera equipped with a Sony FE 90mm Goss lens. Photos were stacked with Helicon Focus (v. 7.6.1) or Zerene Stacker (v. 1.04) and processed in Adobe Photoshop CC2019.

All measurements are in millimeters and were obtained with an Olympus SZX16 stereomicroscope with a Zongyuan CCD industrial camera. Total length is measured without chelicerae. Eye sizes are measured as the maximum diameter from either the dorsal or frontal view. Leg measurements are given as follows: total length (femur, patella, tibia, metatarsus, tarsus). The terminology used in the text and figures follows Ono (1988).

Types of the new species reported here are deposited at the Institute of Zoology, Chinese Academy of Sciences in Beijing.

### ﻿Abbreviations

**ALE** anterior lateral eyes;

**AME** anterior median eyes;

**E** embolus;

**FD** fertilization duct;

**H** hood;

**ITA** intermediate tibial apophysis;

**Mp** median plate;

**PLE** posterior lateral eyes;

**PME** posterior median eyes;

**RTA** retrolateral tibial apophysis;

**S** spermathecae;

**VTA** ventral tibial apophysis.

## ﻿Taxonomy

### ﻿Family Thomisidae Sundevall, 1833

#### ﻿Genus Phrynarachne

Taxon classificationAnimaliaAraneaeThomisidae

Thorell, 1869

B4A66B55-2AA0-5BF3-BD07-9DD92488709E

Phrynarachne Thorell, 1869: 37. For the complete list of references see WSC (2021).

##### Type species.

*Thomisusrugosus* Walckenaer, 1805, from Mauritius

##### Diagnosis.

Large or medium-sized, male is much smaller than the female (1:2 or more). Prosoma nearly as long as wide, with granulations. Eyes small, subequal in size. Fovea inconspicuous. Chelicerae with two promarginal and one retromarginal teeth. Labium longer than wide, sternum oval, male palp with VTA, ITA and RTA; tegulum flat, disk-shaped; tegular ridge present; embolus slender. Female epigynum simple, with a media plate, spermathecae strong sclerotized.

#### ﻿ Phrynarachne brevis

Taxon classificationAnimaliaAraneaeThomisidae

Tang & S. Li, 2010

84D9F28B-51C0-5266-905F-67D5735F244E

**Figure 1.**
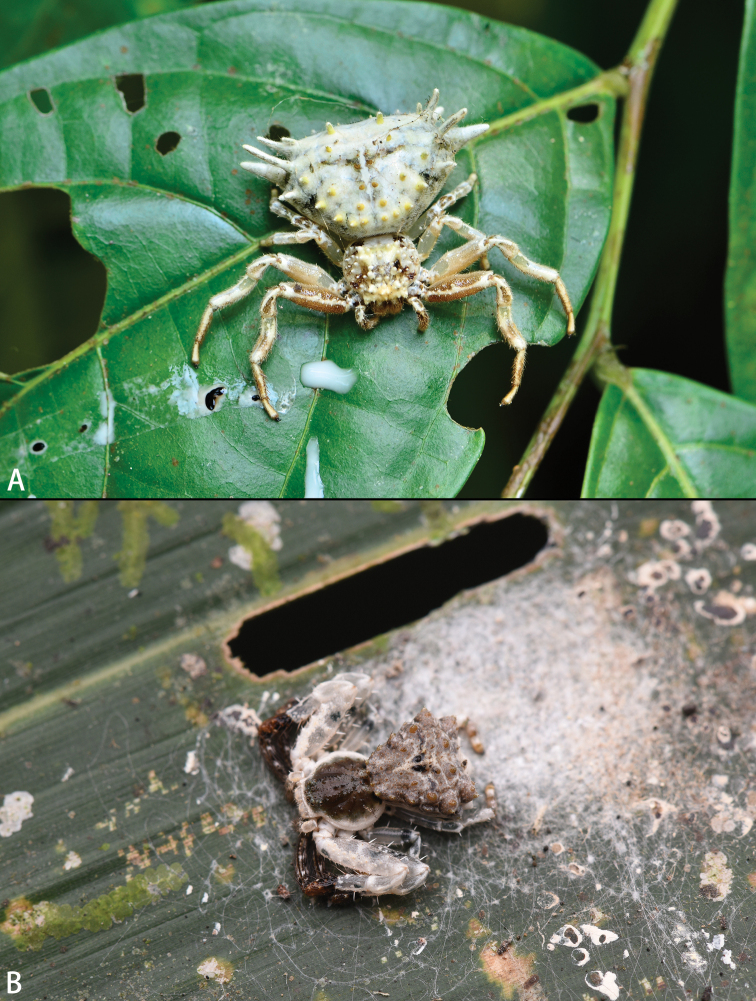
*Phrynarachne* spp., live **A***P.brevis*, adult female **B***P.xuxiake* sp. nov., juvenile. Photos by Chao Wu (**A**) and Fan Gao (**B**).

Figs 1A , 6 , 18A , 21

Phrynarachne brevis Tang & Li, 2010: 49, figs 35A–D, 36A, B (♂).

##### Type material.

***Holotype***: ♂ (IZCASAr18535), **China: *Yunnan***: Xishuangbanna, Mengla County, Menglun Town, Menglun Nature Reserve, Bamboo plantation near G213 roadside, 21.8940°N, 101.2823°E, 580 m elev., 3.XII.2009, Guo Tang and Zhiyuan Yao leg., examined.

##### Other material examined.

1♀ (IZCAS-Ar41642), **China: *Yunnan***: Xishuangbanna, Jinghong City, Mengla County, Menglun Town, Menglun Nature Reserve, Bamboo plantation, 21.9008°N, 101.2822°E, 597 m elev., 9.V.2019, Zhigang Chen leg.

##### Diagnosis.

See diagnosis of *P.dreepy* sp. nov.

##### Description.

**Female** (Figs 1A, 6, 18A). Total length 16.53, carapace 6.62 long, 7.09 wide, yellow brown with brown pattern and granulations dorsally. With large projection between ALE and PLE. Eye sizes and interdistances: ALE 0.26, AME 0.23, PLE 0.25, PME 0.22; ALE–AME 0.51, AME–AME 0.90, PLE–PME 1.62, PME–PME 1.02. Chelicerae brown, with two promarginal and one retromarginal teeth; gnathocoxae, labium dark yellow, labium 1.52 long, 1.18 wide. Sternum yellow. Legs brown, femora I and II with dense, varying-sized tubercles; tibiae and metatarsi I, II with dense asymmetrical ventral spines (I, tibia 12, metatarsus 30; II, tibia 10, metatarsus 30). Leg measurements: I 17.25 (5.74, 6.31, 3.10, 2.10), II 17.61 (5.66, 6.61, 3.30, 2.04), III 12.30 (3.67, 5.74, 1.50, 1.39), IV 10.26 (3.31, 4.12, 1.46, 1.37). Opisthosoma dorsally light yellow, each side with 22 long tubercles, middle with pair of black markings.

Epigyne (Fig. 6) with M-shaped sclerotized margins; median plate obvious, with a posterior hood, anterior edge recurved and posterior edge almost straight, the ratio of length to width is 11:3; copulatory opening obvious; spermathecae kidney-shaped, the ratio of anterior edge to posterior edge length is 1:1. Fertilization duct transverse.

**Male.** See Tang and Li (2010).

##### Distribution.

China (Yunnan).

##### Notes.

The female is described here for the first time.

#### ﻿ Phrynarachne ceylonica

Taxon classificationAnimaliaAraneaeThomisidae

(O. Pickard-Cambridge, 1884)

C795BEB5-5F03-510B-9151-57100B931A25

Figs 2 , 21

Ornithoscatoides ceylonica O. Pickard-Cambridge, 1884: 201, pl. 15, fig. 3. For the complete list of references see WSC (2021).

##### Type material.

***Syntypes*** 2♀, “Ceylon, G.H.K. Thwaites leg.”, Hope Department of Entomology, Oxford, UK, not examined; *O.nigra* O. Pickard-Cambridge, 1884: ***Syntypes*** 2♂, “Ceylon and India, G.H.K. Thwaites leg.”, Hope Department of Entomology, Oxford, UK, not examined.

**Figure 2.**
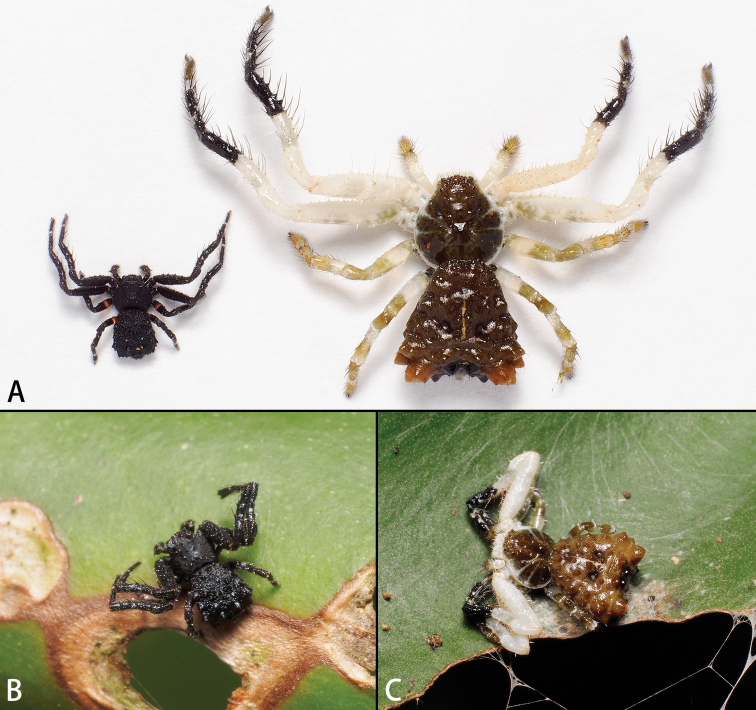
*Phrynarachneceylonica***A** adult male (left) and female (right) **B** male live **C** female live. Photos by Peter Koomen.

##### Other material examined.

2♂ (IZCAS), Xishuangbanna, Jinghong City, Mengla County, Menglun Town, Menglun Nature Reserve, primary tropical seasonal rain forest, 21.9598°N, 101.2035°E, 822 m elev., 8.VIII.2007, Guo Zheng leg.; 1♂ (IZCAS), Xishuangbanna, Jinghong City, Mengla County, Menglun Town, Menglun Nature Reserve, *Paramicheliabaillonii* plantation (about 20 years old), 21.9129°N, 101.2674°E, 556 m elev., 18.VII.2007, Guo Zheng leg.; 2♂ (IZCAS), Xishuangbanna, Jinghong City, Mengla County, Menglun Town, Menglun Nature Reserve, secondary tropical seasonal moist forest, 21.9120°N, 101.2823°E, 645 m elev., 27.VII.2007, Guo Zheng leg.; 1♂ (IZCAS), Xishuangbanna, Jinghong City, Mengla County, Menglun Town, Menglun Nature Reserve, *Anogeissusacuminata* plantation (about 20 yr.), 21.8999°N, 101.2802°E, 611 m elev., 19.VIII.2007, Guo Zheng leg.; 2♀ (IZCAS), Xishuangbanna, Jinghong City, Mengla County, Menglun Town, Menglun Nature Reserve, secondary tropical seasonal moist forest, 21.9065°N, 101.2802°E, 612 m elev., 10.VIII.2007, Guo Zheng leg.

##### Distribution.

Asia: from India and Sri Lanka to Japan, south to Indonesia. In China is known from Guangxi, Taiwan, and Yunnan.

#### ﻿ Phrynarachne dreepy

Taxon classificationAnimaliaAraneaeThomisidae

Lin & S. Li sp. nov.

8DA2EDFB-3305-560C-A44C-BB51443DA973

http://zoobank.org/E0BC4600-8F1E-403A-BBDD-C37EDE540845

**Figure 3.**
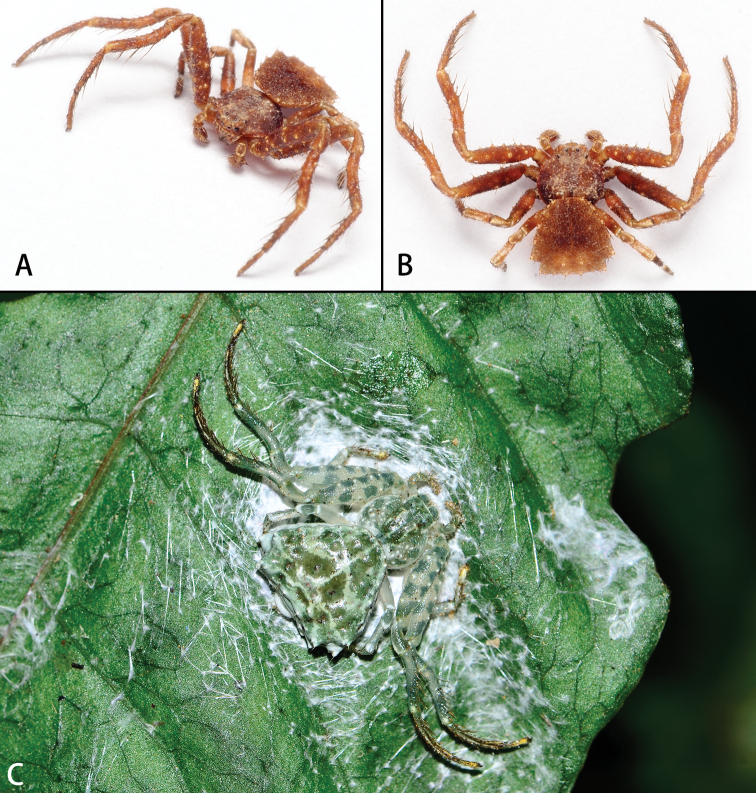
*Phrynarachnedreepy* sp. nov. **A, B** male **C** female life. Photos by Peter Koomen (**A, B**) and Chao Wu (**C**).

Figs 3 , 7 , 8 , 18C, D , 21

##### Type material.

***Holotype***: ♂ (IZCAS-Ar41643), **China: *Yunnan***: Xishuangbanna, Jinghong City, Mengla County, Menglun Town, Menglun Nature Reserve, 21.9768°N, 101.2010°E, 814 m elev., 17.VIII.2011, Guo Zheng leg.; ***Paratypes***: 2♀ (IZCAS-Ar41644, Ar41645), **China: *Yunnan***: Xishuangbanna, Jinghong City, Mengla County, Menglun Town, Menglun Nature Reserve, Xishuangbanna Tropical Botanic Garden, 21.9277°N, 101.2622°E, 552 m elev., VIII.2019, Long Yu leg.; 1♂ (IZCAS-Ar41646), same data as holotype, but 21.9502°N, 101.2010°E, 814 m elev., 18.VIII.2011; 2♂ (IZCAS-Ar41647, Ar41648), Xishuangbanna, Jinghong City, Guanping Town, Shiwudui, 22.2280°N, 100.8894°E, 888 m elev., 20.VII.2012, Qingyuan Zhao and Zhigang Chen leg.

##### Etymology.

The species is named after *Dreepy*, a fictional character from Pokémon Sword and Shield, who has a triangular head that is reminiscent of the opisthosoma of the new species; noun (name) in apposition.

##### Diagnosis.

*Phrynarachnedreepy* sp. nov. is similar to *P.brevis* in that males have a long RTA; in females the epigyne has sclerotized margins and the posterior edge of the median plate has a depression. However, males of *P.dreepy* sp. nov. can be easily distinguished by the long VTA (vs short VTA in *P.brevis*), the length of embolus to the length of the embolus base (7:1 vs 18:1 in *P.brevis*), and the embolus separate from the tegulum (vs close to the tegulum in *P.brevis*). Females can be separated from *P.brevis* by the short, triangular tubercles on the abdomen (vs long, slender tubercles in *P.brevis*), the straight anterior edge of median plate (vs recurved in *P.brevis*), and the procurved posterior edge of the median plate (vs almost straight in *P.brevis*).

##### Description.

**Male** (Figs 3A, B, 7, 18C), ***holotype***: total length 2.26, carapace 1.04 long, 1.02 wide, yellow-brown, with white tubercles. Eye sizes and interdistances: ALE 0.09, AME 0.07, PLE 0.07, PME 0.06; ALE–AME 0.05, AME–AME 0.07, PLE–PME 0.09, PME–PME 0.11. Chelicerae brown, with two promarginal teeth and one retromarginal tooth; gnathocoxae, yellow-brown, labium brown, 0.23 long, 0.18 wide. Sternum yellow-brown. Legs yellow-brown, femora I ang II with dense, varying-sized tubercles; tibiae and metatarsi I, II with pairs of ventral spines (I, tibia 6, metatarsus 8; II, tibia 6, metatarsus 6). Leg measurements: I 3.85 (1.21, 1.38, 0.76, 0.50), II 3.78 (1.22, 1.34, 0.73, 0.49), III 1.72 (0.55, 0.60, 0.27, 0.30), IV 1.52 (0.51, 0.52, 0.23, 0.26). Leg formula: 1234. Opisthosoma dark brown, each side with 18 tubercles, each with a clavate seta.

Male palp (Fig. 3A, B). Tibia brown, VTA club-shaped; RTA long, the length of VTA to the length of RTA is 3:1. Cymbium brown. Tegulum flat, disk-shaped, with a tegular ridge. Embolus spiraled, thin, separated from tegulum; the length of embolus to the length of embolus base 7:1.

**Female** (Figs 3C, 8, 18D) one ***paratype***: total length 8.45, carapace 3.77 long, 4.02 wide, pale yellow, green when alive. Eye sizes and interdistances: ALE 0.22, AME 0.12, PLE 0.20, PME 0.15; ALE–AME 0.18, AME–AME 0.24, PLE–PME 0.28, PME–PME 1.02. Chelicerae brown, with two promarginal teeth and one retromarginal tooth; gnathocoxae, labium yellow, labium 0.86 long, 0.63 wide. Sternum yellow. Legs pale yellow, femora I and II with dense, varying-sized tubercles; tibiae and metatarsi I, II with dense asymmetrical ventral spines (I, tibia 25, metatarsus 102; II, tibia 20, metatarsus 83). Leg measurements: I 13.87 (4.26, 4.94, 3.10, 1.57), II 13.99 (4.40, 4.81, 3.16, 1.62), III 7.17 (2.36, 2.67, 1.12, 1.02), IV 6.74 (2.49, 2.57, 0.82, 0.86). Leg formula: 2134. Opisthosoma pale green, each side with 13 triangular tubercles, each with a clavate seta.

Epigyne (Fig. 8) with sclerotized margins; median plate almost rectangular, hood absent, anterior edge straight, posterior edge slightly recurved, the ratio of length to width is 4:1; copulatory opening inconspicuous; spermathecae kidney-shaped, the ratio of anterior edge to posterior edge length is 2:1. Fertilization duct transverse.

##### Distribution.

Known only from the type locality.

#### ﻿ Phrynarachne huangshanensis

Taxon classificationAnimaliaAraneaeThomisidae

Li, Chen & Song, 1985

49F5B061-59A0-5C34-A3E6-1C9CB4FEDE28

Figs 5 , 9 , 10 , 17A, C , 18B , 21

Phrynarachne huangshanensis Li et al., 1985: 73, figs 1, 2. For the complete list of references see WSC (2021).

##### Type material.

***Holotype***: ♀ (IZCAS-Ar41649), **China: *Anhui***: Huangshan City, Huangshan District, Zhaixi Village, 30.0580°N, 118.1664°E, 423 m elev., 14.VI.1982, Youcai Li, Fayang Chen and Daxiang Song leg., examined.

##### Other material examined.

1♂(IZCAS-Ar416450), **China: *Anhui***: Huangshan City, Tangkou Town, Houyuan, ravine, 30.0735°N, 118.1522°E, 470 m elev., IX.2018, Long Yu leg.; 2♂(IZCAS-Ar41651, Ar41652), **China: *Anhui***: Huangshan City, Tangkou town, Fangcunxin Village, ravine, shrub with broad leaves, 30.0457°N, 118.1606°E, 430±8 m elev., 5.IX.2019, Long Yu leg.; 3♂ (IZCAS-Ar41653–Ar41655), **China: *Anhui***: Huangshan City, Tangkou town, Fangcun Village, shrub with broad leaves, 30.0302°N, 118.1822°E, 356±6 m elev., 5.IX.2019, Long Yu leg; 5♀(IZCAS-Ar41656–Ar41660), **China: *Anhui***: Huangshan City, Tangkou Town, Fangcunxin Village, ravine, 30.0501°N, 118.1854°E, 450 m elev., IX.2018, Long Yu leg.

##### Diagnosis.

Males of *Phrynarachnehuangshanensis* can be distinguished from those of *P.mammillata* by the ratio of the length of the embolus to the length of the embolus base (7:1 in *P.huangshanensis* vs 10:1 in *P.mammillata*), and the ratio of the length of the RTA to the length of the VTA (3:1 in *P.huangshanensis* vs 2:1 in *P.mammillata*). Females can be differentiated by the length to width ratio of the median plate (3:1 in *P.huangshanensis* vs 5:1 in *P.mammillata*), and the V-shaped median plate (vs M-shaped in *P.mammillata*).

##### Description.

**Male** (Figs 5A, 9, 18B): total length 2.45, carapace 1.10 long, 1.14 wide, dark brown with long setae. Opisthosoma brown in middle, with some tubercles, each with a clavate seta. A pair of white lines from PLE to fovea. Eye sizes and interdistances: ALE 0.09, AME 0.06, PLE 0.07, PME 0.04; ALE–AME 0.05, AME–AME 0.11, PLE–PME 0.13, PME–PME 0.15. Chelicerae brown, with two promarginal teeth and one retromarginal tooth; gnathocoxae, labium dark brown, labium 0.20 long, 0.21 wide. Sternum black. Legs black, femora I and II with dense, varying-sized tubercles, tibiae and metatarsi I, II with pairs of ventral spines (I, tibia 6, metatarsus 6; II, tibia 6, metatarsus 6); femora III, IV with white stripe. Leg measurements: I 3.54 (1.13, 1.23, 0.66, 0.52), II 3.50 (1.18, 1.22, 0.60, 0.50), III 1.69 (0.55, 0.56, 0.26, 0.32), IV 2.08 (0.73, 0.72, 0.28, 0.35). Leg formula: 1234. Opisthosoma dorsally dark brown, each side with 17 tubercles, each with a clavate seta, center with a pair of yellow markings.

Male palp (Fig. 9). Tibia brown, VTA club-shaped; RTA long, the length ratio of VTA to RTA is 3:1. Cymbium black. Tegulum flat, disk-shaped, with a tegular ridge. Embolus spiral, thin, the length ratio of the embolus to the embolus base is 7:1.

**Female.** See Li et al. (1985).

##### Distribution.

China (Anhui).

##### Notes.

The male is described for the first time here.

#### ﻿ Phrynarachne katoi

Taxon classificationAnimaliaAraneaeThomisidae

Chikuni, 1955

3DEA28A6-58A9-5103-9AD4-BA468350FE8C

Figs 4 , 21

Phrynarachne katoi Chikuni, 1955: 35, figs 4A–G, pl. 1. For the complete list of references see WSC (2021).

##### Type material.

***Holotype*** 1♀ (Collection of Kyukichi Kishida, Tokyo), from Tojigami, Daisan-ku, Kawajimura, Iida-shi, Shimoina-gun, Nagano Pref., 470 m elev., 7.IX.1953, S. Sekigawa leg., not examined.

##### Other material examined.

1♂1♀ (IZCAS), **China: *Anhui***: Huangshan City, Xiuning County, Mount Qiyun, 29.8186°N, 118.0294°E, 24.X.2021, Fan Gao leg.

##### Distribution.

China, Korea, and Japan. In China it is known from Anhui, Zhejiang.

**Figure 4.**
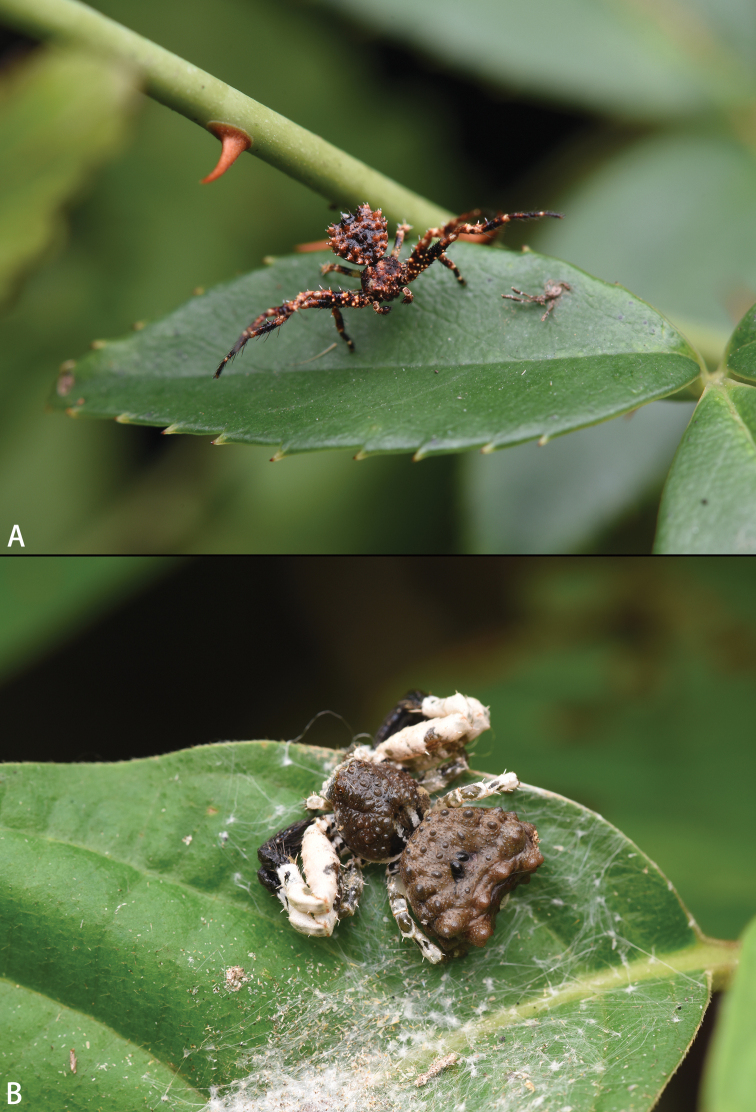
*Phrynarachnekatoi*, live **A** male **B** female. Photos by Fan Gao.

**Figure 5.**
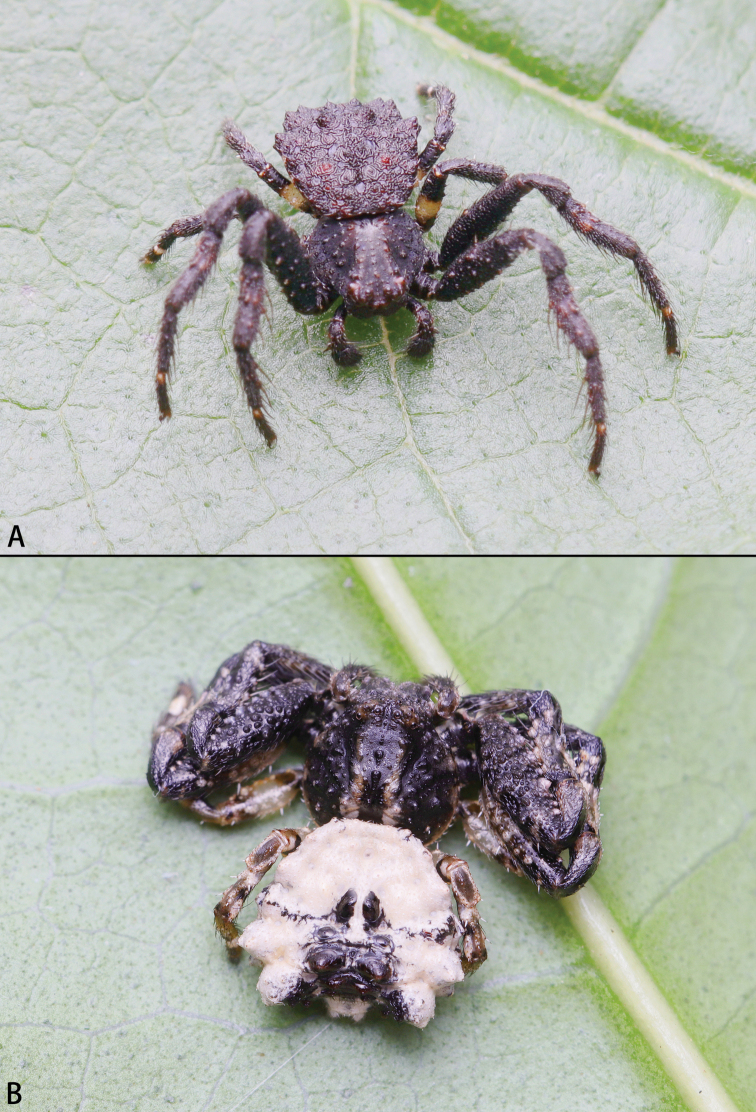
*Phrynarachnehuangshanensis*, live **A** male **B** female. Photos by Ruiyang Wang.

**Figure 6.**
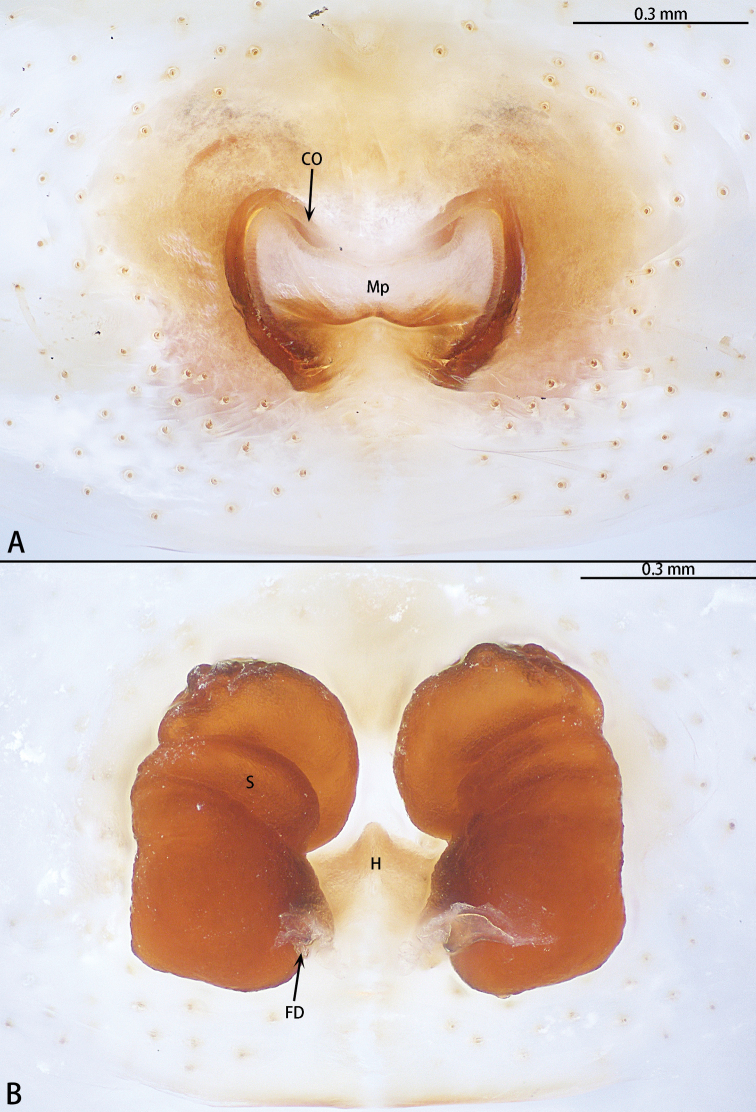
*Phrynarachnebrevis*, female **A** epigyne, ventral **B** vulva, dorsal.

**Figure 7.**
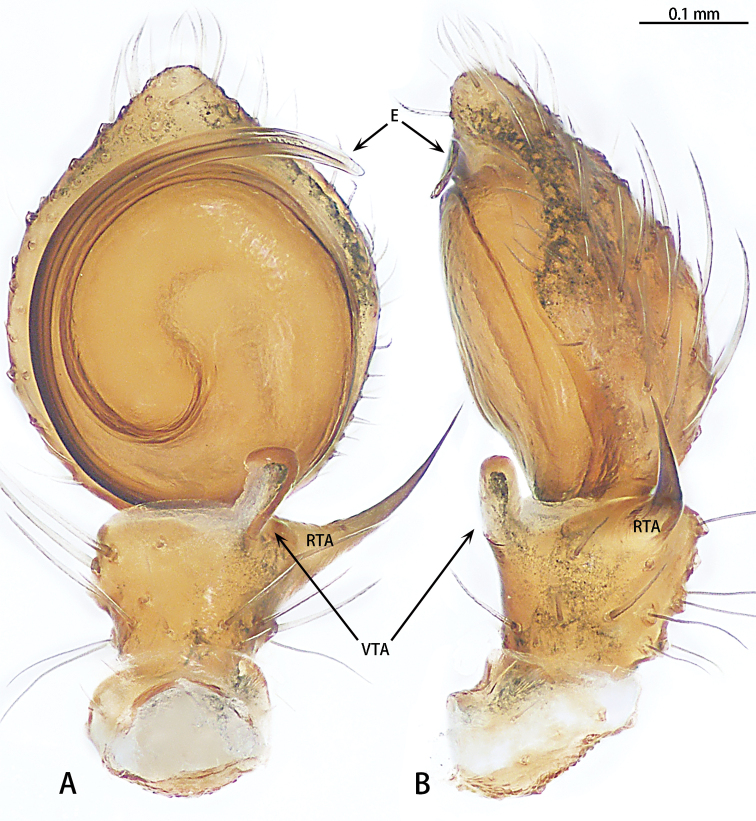
*Phrynarachnedreepy* sp. nov., holotype male, left palp **A** ventral **B** retrolateral.

#### ﻿ Phrynarachne lancea

Taxon classificationAnimaliaAraneaeThomisidae

Tang & S. Li, 2010

602C5478-E2AA-53CF-83C6-452DF365605B

Figs 11 , 19A , 21

Phrynarachne lancea Tang & Li, 2010: 53, figs 37A–D, 38A, B.

##### Type material.

***Holotype***: ♂ (IZCAS-Ar18536), **China: *Yunnan***: Xishuangbanna, Jinghong City, Mengla County, Menglun Town, Menglun Nature Reserve, Tropical seasonal rainforest, 21.9368°N, 101.2701°E, 558 m elev., 1.XII.2009, Guo Tang and Zhiyuan Yao leg., examined. ***Paratype***: 1♂(IZCAS-Ar18537), **China: *Yunnan***: Xishuangbanna, Jinghong City, Mengla County, Menglun Town, Menglun Nature Reserve, *Anogeissusacuminata* plantation (about 20 years old), 21.8970°N, 101.2846°E, 609 m elev., 27.XII.2009, Guo Tang and Zhiyuan Yao leg. examined.

##### Other material examined.

3♀(IZCAS-Ar41661–Ar41663), **China: *Yunnan***: Xishuangbanna, Jinghong City, Mengla County, Xishuangbanna Tropical Botanic Garden, Rainforest Valley, 21.9277°N, 101.2622°E, 552 m elev., III.2018, Yu Long leg.; 2♀ (IZCAS-Ar41664, Ar41665), same data as above, but II.2019; 3♂(IZCAS-Ar41666–Ar41668), same data as above, but V.2019.

##### Diagnosis.

*Phrynarachnelancea* males can be easily distinguished from other species by the wide, spear-shaped RTA. Females of *P.lancea* are similar to *P.mammillata* in having an M-shaped median plate and kidney-shaped spermathecae. However, *P.lancea* can be distinguished by the length to width ratio of the median plate (7:1 in *P.lancea* vs 4:1 in *P.mammillata*), the straight posterior edge of the median plate (vs procurved in *P.mammillata*), the posterior edge of the spermathecae shorter than the anterior edge (vs of equal length in *P.mammillata*), and the longitudinal fertilization ducts (vs transverse in *P.mammillata*).

##### Description.

**Female** (Figs 11, 19A): total length 16.49, carapace 6.53 long, 6.82 wide, white, posterior edge black. Eye sizes and interdistances: ALE 0.21, AME 0.20, PLE 0.24, PME 0.21; ALE–AME 0.14, AME–AME 0.25, PLE–PME 0.33, PME–PME 0.28. Chelicerae white, with two promarginal teeth and one retromarginal tooth; gnathocoxae white with black pattern, labium black, 0.88 long, 0.83 wide. Sternum white. Legs white with black markings, femora I and II with dense, varying-sized tubercles; tibiae and metatarsi I, II with dense asymmetrical ventral spines (I, tibia 28, metatarsus 75; II, tibia 26, metatarsus 68). Leg measurements: I 12.45 (4.23, 4.49, 2.41, 1.32), II 12.15 (4.15, 4.41, 2.31, 1.28), III 6.25 (2.12, 2.38, 0.98, 0.87), IV 5.90 (2.18, 2.00, 0.92, 0.80). Leg formula: 1234. Opisthosoma white, posterior grey, with four obvious brown tubercles.

Epigyne (Fig. 11) with sclerotized margins inconspicuous, M-shaped; median plate M-shaped, hood absent, anterior and posterior edges recurved, the ratio of length to width is 7:1; copulatory opening inconspicuous; spermathecae kidney-shaped, the ratio of anterior edge to posterior edge length is 3:1. Fertilization duct longitudinal.

**Male.** See Tang and Li (2010).

##### Distribution.

China (Yunnan).

##### Notes.

The female is reported here for the first time.

#### ﻿ Phrynarachne mammillata

Taxon classificationAnimaliaAraneaeThomisidae

Song, 1990

85C87164-6611-5C2C-9EC3-A1E8547FCEFB

Figs 12 , 13 , 17B, D , 19B , 21

Phrynarachne mammillata Song in Song & Cha﻿﻿﻿﻿i, 1990: 364, fig. 1A–D.For the complete list of references see WSC (2021).

##### Type material.

***Holotype***: ♀ (IZCAS-Ar9358), **China: *Guizhou***: Tongren City, Jiangkou County, Fanjing Mountain, 10.VII.1988, examined.

**Figure 8.**
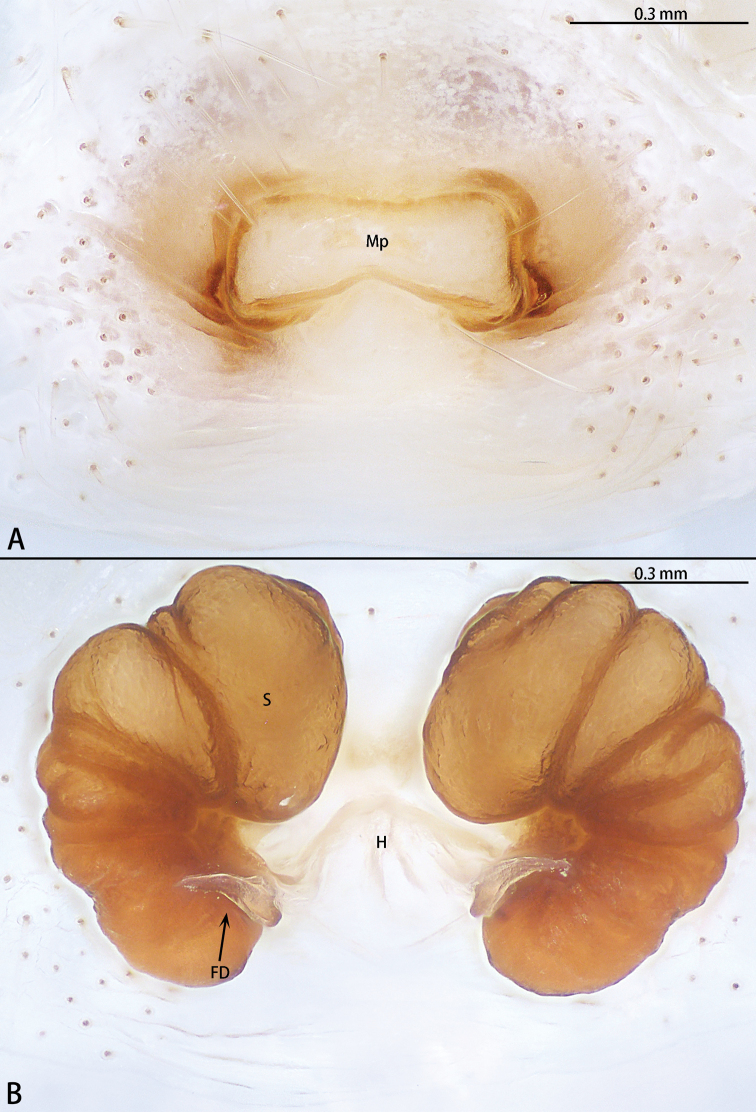
*Phrynarachnedreepy* sp. nov., patatype female **A** epigyne, ventral **B** vulva, dorsal.

**Figure 9.**
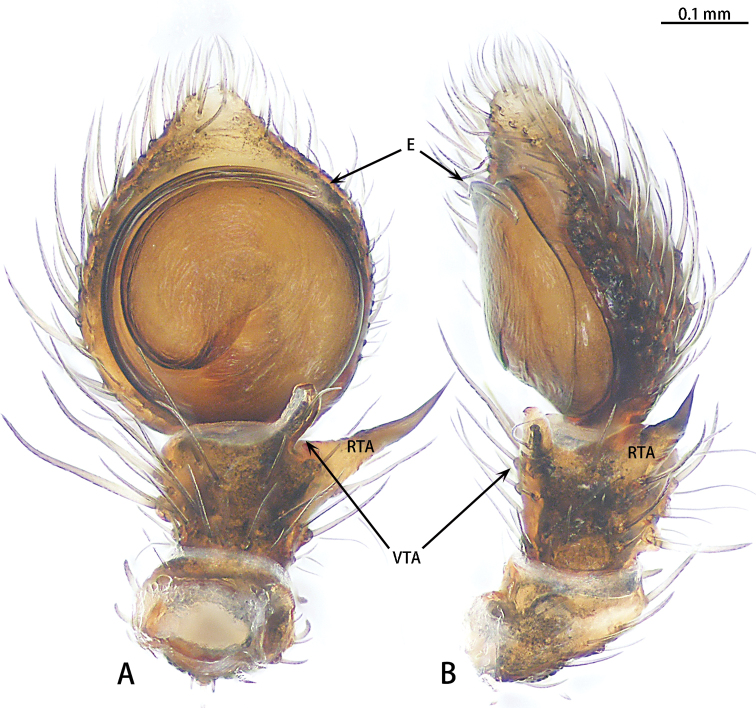
*Phrynarachnehuangshanensis*, male left palp **A** ventral **B** retrolateral.

##### Other material examined.

2♀ (IZCAS-Ar41669, Ar41670), **China, *Yunnan***: Xishuangbanna, Jinghong City, Mengla County, Xishuangbanna Tropical Botanic Garden, Rainforest Valley, 21.927745°N, 101.262194°E, 552 m elev., 2014/VII, Yu Long leg.; 1♂ (IZCAS-Ar41671), Xishuangbanna, Jinghong City, Guanping Town, Shiwudui, 22.2280°N, 100.8894°E, 888 m elev., 20.VII.2012, Qingyuan Zhao and Zhigang Chen leg.

##### Diagnosis.

See diagnosis of *Phrynarachnehuangshanensis*.

##### Description.

**Male** (Figs 12, 19B): total length 1.82, carapace 0.90 long, 0.97 wide, dark brown, cephalic region yellow-brown. Eye sizes and interdistances: ALE 0.09, AME 0.06, PLE 0.07, PME 0.06; ALE–AME 0.05, AME–AME 0.07, PLE–PME 0.12, PME–PME 0.12. Chelicerae brown, with two promarginal teeth and one retromarginal tooth; gnathocoxae, labium dark brown, labium 0.15 long, 0.19 wide. Sternum brown. Legs black, femora I and II with dense, varying-sized tubercles; tibiae and metatarsi I, II with pairs of ventral spines (I, tibia 6, metatarsus 6; II, tibia 6, metatarsus 6), femora III, IV with broad white pattern. Leg measurements: I 2.51 (0.83, 0.83, 0.51, 0.34), II 2.51 (0. 81, 0.86, 0.46, 0.38), III 1.47 (0.47, 0.48, 0.26, 0.26), IV 1.36 (0.48, 0.42, 0.24, 0.22). Leg formula: 1 = 234. Opisthosoma dark brown with yellow-brown spots, each side with 18 tubercles, yellow-brown tubercles, each with a clavate seta.

**Figure 10.**
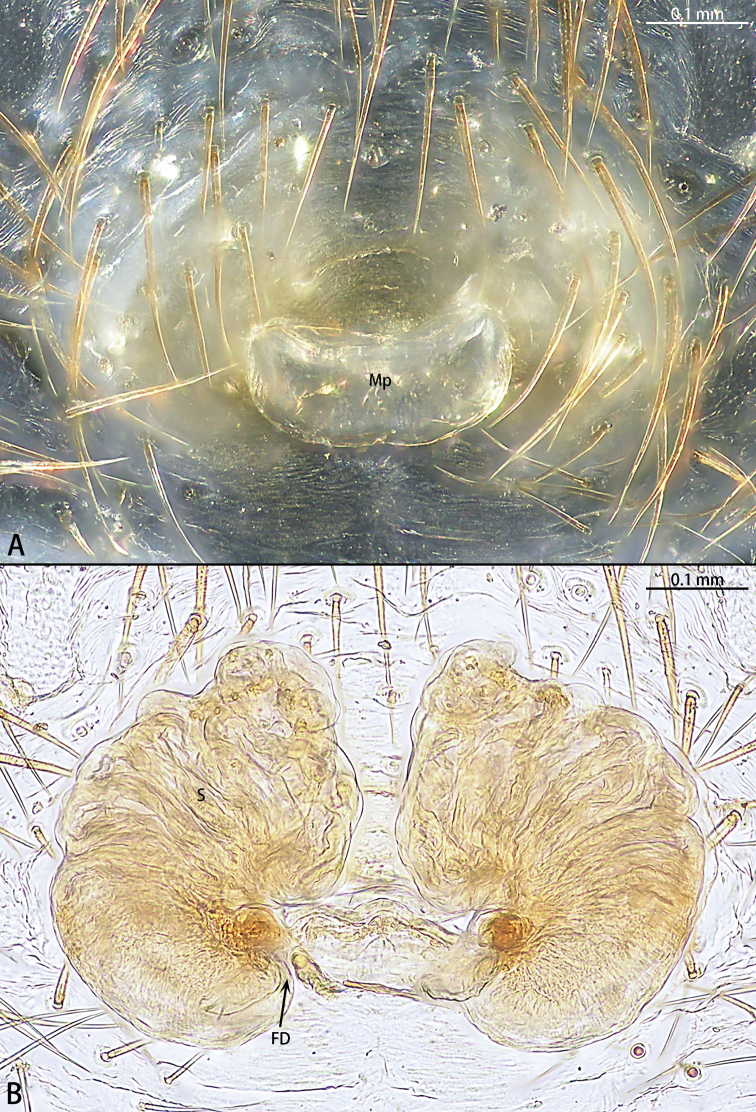
*Phrynarachnehuangshanensis*, holotype female **A** epigyne, ventral **B** vulva, dorsal.

Male palp (Fig. 12). Tibia brown, VTA club-shaped; RTA long, the length ratio of VTA to RTA is 2:1. Cymbium yellow to brown. Tegulum flat, disk-shaped, with a tegular ridge. Embolus spiraled, thin, the length ratio of embolus to embolus base is 10:1.

**Female.** See Song and Chai (1990).

##### Distribution.

China (Guizhou, Yunnan).

##### Notes.

The male is reported here for the first time.

#### ﻿ Phrynarachne sinensis

Taxon classificationAnimaliaAraneaeThomisidae

Peng, Yin & Kim, 2004 nomen dubium

DD847B39-ADD2-5DD4-9705-727527BB2841

Phrynarachne sinensis Peng et al., 2004: 21, figs 1–3; Yin et al. 2012: 1265, fig. 680a–c.

##### Type material.

***Holotype***: ♀ (College of Life Sciences, Hunan Normal University), China (Wang-101), no detailed data, lost, not examined.

##### Notes.

The lost type specimen, lack of clear figures of the holotype, and the vague distributional information make further study of the taxonomy of this species impossible. We treat it as *nomen dubium*.

#### ﻿ Phrynarachne xuxiake

Taxon classificationAnimaliaAraneaeThomisidae

Lin & S. Li sp. nov.

E6B9BD64-3EC7-50B6-B099-DEA9E92D1040

http://zoobank.org/BB4750BD-1DC5-4B3C-BF29-2F26118EA68D

Figs 1B , 14 , 19C , 21

##### Type material.

***Holotype***: ♀ (IZCAS-Ar41672), **China: *Anhui***: Huangshan City, Tangkou Town, Fangcunxin Village, ravine, 30.0501°N, 118.1854°E, 450 m elev., IX.2018, Long Yu leg.

##### Etymology.

The species is named after Xu Xiake, a Chinese travel writer and geographer of the Ming dynasty; noun (name) in apposition.

##### Diagnosis.

Females of *Phrynarachnexuxiake* sp. nov. are similar to *P.katoi* but can be distinguished by the length to width ratio of the median plate (3:1 in *P.xuxiake* vs 5:1 in *P.katoi*) and by the rectangular median plate with its posterior edge straight (vs dumbbell-shaped with procurved posterior edge in *P.katoi*).

##### Description.

**Female** (Figs 14, 19C), ***holotype***: total length 8.78, carapace 3.84 long, 4.45 wide, dark brown with long setae. Eye sizes and interdistances: ALE 0.22, AME 0.17, PLE 0.18, PME 0.15; ALE–AME 0.18, AME–AME 0.24, PLE–PME 0.28, PME–PME 0.28. Chelicerae white, with two promarginal teeth and one retromarginal tooth; gnathocoxae, labium black, labium 0.84 long, 0.74 wide. Sternum black. Legs yellow, femora I and II with dense, varying-sized tubercles; tibiae and metatarsi of I, II with dense ventral spines (I, tibia 26, metatarsus 108; II, tibia 21, metatarsus 89). Leg measurements: I 15.11 (4.17, 4.92, 3.70, 2.32), II 13.93 (4.02, 4.68, 3.13, 2.10), III 6.94 (2.12, 2.71, 1.05, 1.06), IV 6.63 (2.39, 2.54, 0.76, 0.94). Leg formula: 1234. Opisthosoma brown, each side with 19 blunt tubercles, each with a clavate seta, with a pair of black markings medially.

**Figure 11.**
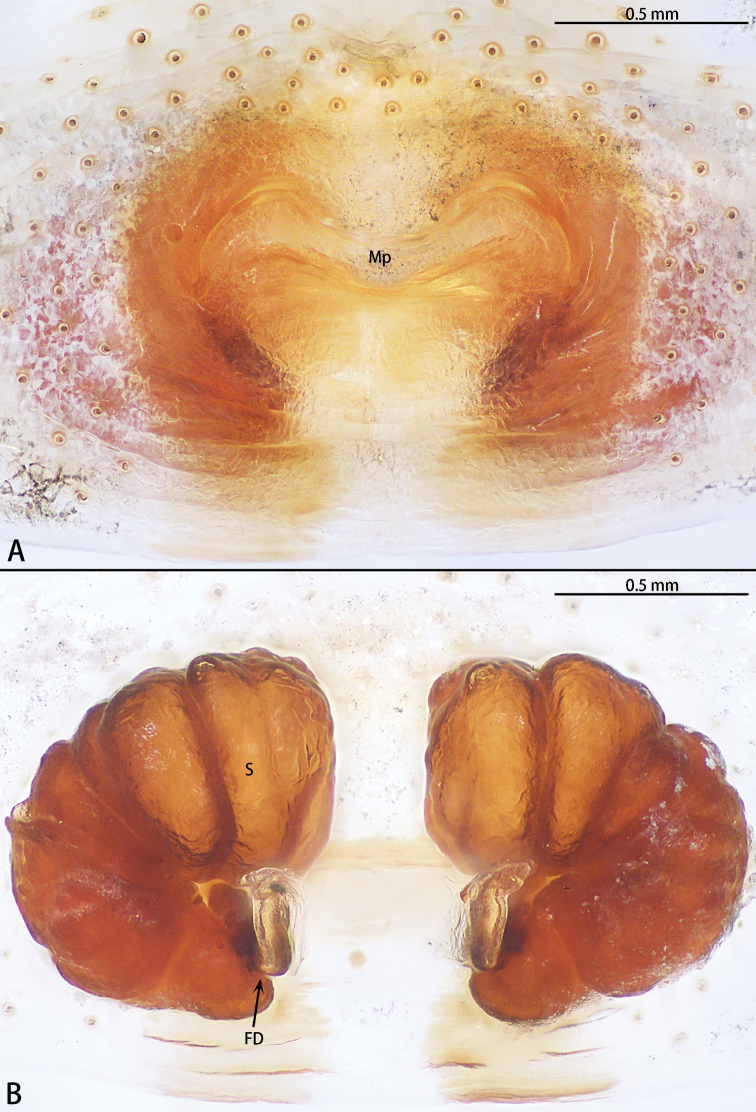
*Phrynarachnelancea*, female **A** epigyne, ventral **B** vulva, dorsal.

**Figure 12.**
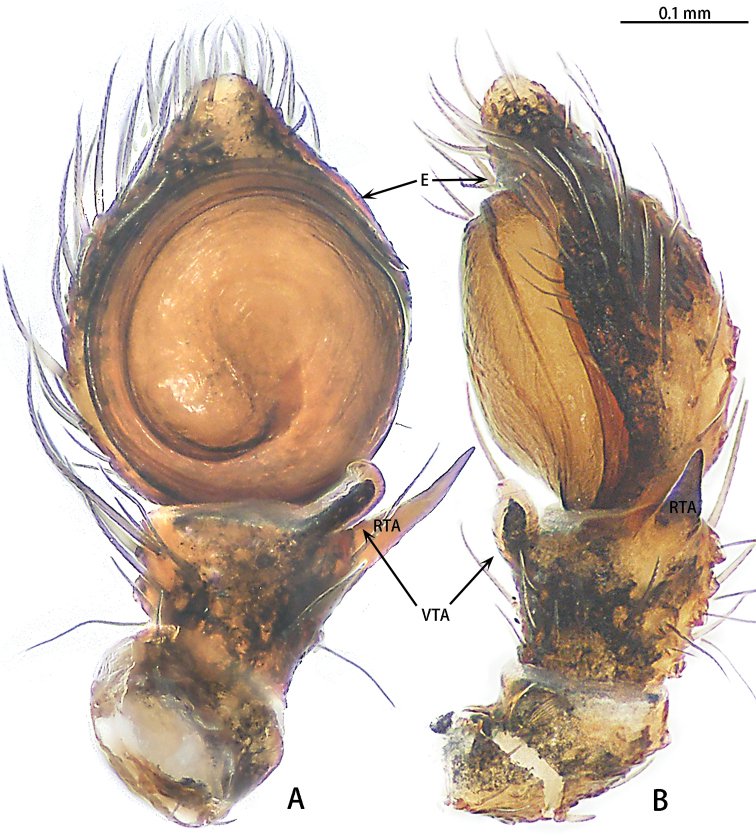
*Phrynarachnemammillata*, male left palp **A** ventral **B** retrolateral.

Epigyne (Fig. 14). Sclerotized margins inconspicuous, M-shaped; median plate obvious, with a posterior hood, anterior edge recurved and posterior edge almost straight, the ratio of width to length is 3:1; copulatory opening inconspicuous; Spermathecae kidney-shaped, the ratio of anterior edge to posterior edge length is 3:2. Fertilization duct transverse.

##### Distribution.

Known only from the type locality.

**Figure 13.**
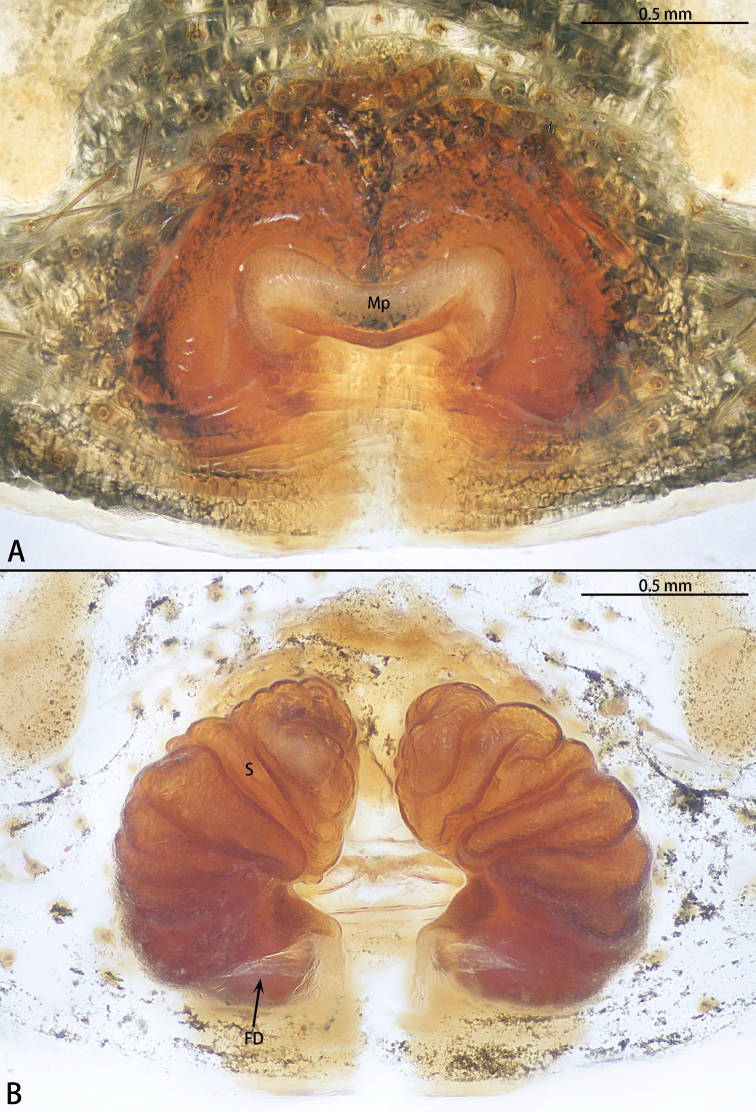
*Phrynarachnemammillata* Song, 1990, female **A** epigyne, ventral **B** vulva, dorsal.

**Figure 14.**
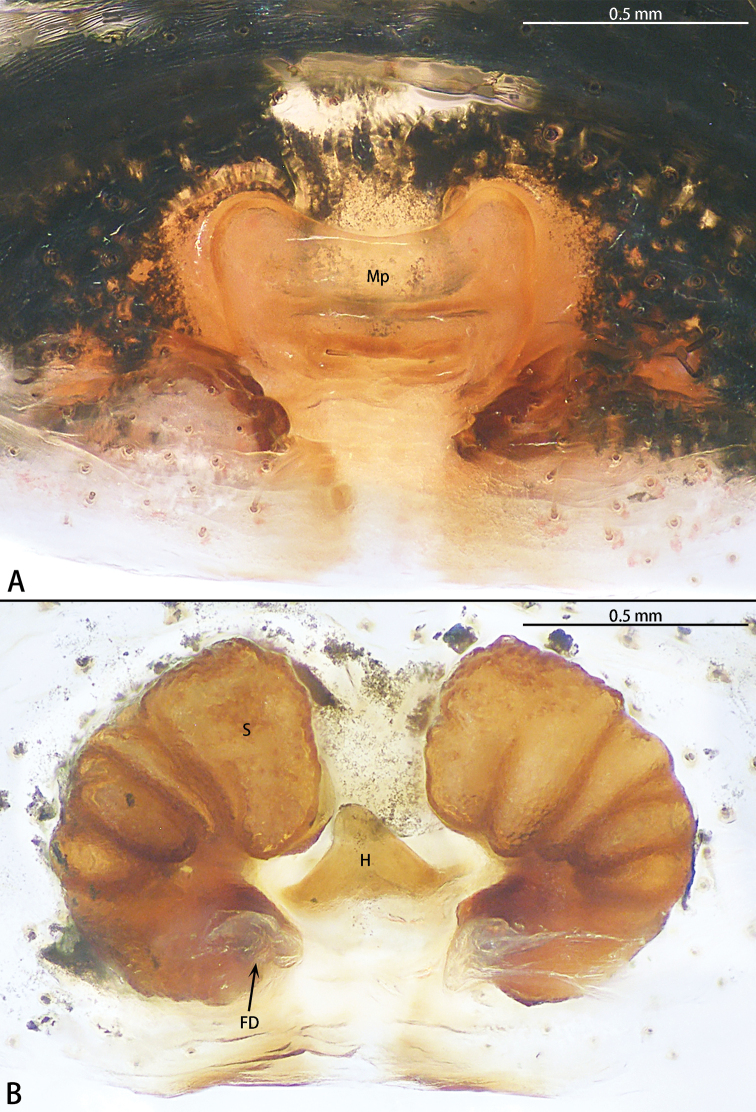
*Phrynarachnexuxiake* sp. nov., holotype female **A** epigyne, ventral **B** vulva, dorsal.

**Figure 15.**
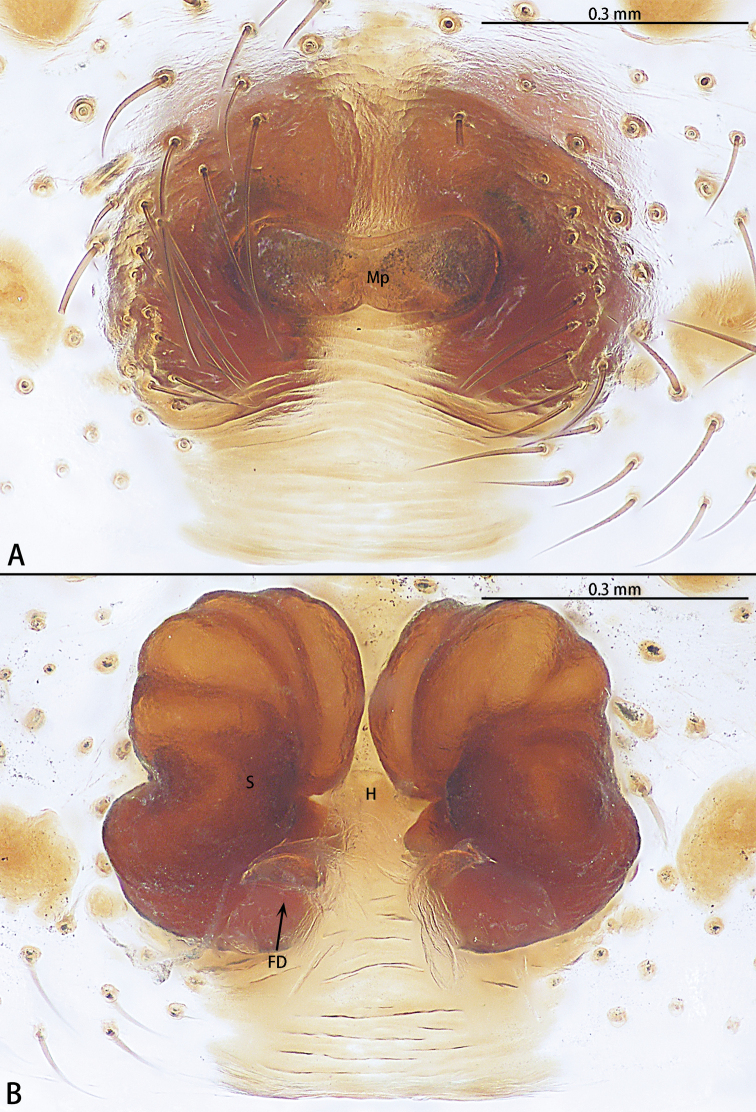
*Phrynarachneyunhui* sp. nov., holotype female **A** epigyne, ventral **B** vulva, dorsal.

#### ﻿ Phrynarachne yunhui

Taxon classificationAnimaliaAraneaeThomisidae

Lin & S. Li sp. nov.

7AA0EA7D-4FA0-554E-B893-671366288DF9

http://zoobank.org/51542955-2CCB-4A0B-881A-FED561B28CB8

Figs 15 , 19D , 21

##### Type material.

***Holotype***: ♀ (IZCAS-Ar41673), **China: *Hainan***: Ledong County, Jianfengling Nature Reserve, Mingfenggu, 18.7417°N, 108.8417°E, 989 m elev., 1.VII.2020, Yunhu Mo leg.

##### Etymology.

The species is named after Mr Yunhu Mo, who collected the holotype; noun (name) in genitive case.

##### Diagnosis.

Females of *Phrynarachneyunhui* sp. nov. are similar to *P.mammillata* in having the anterior edge and posterior edges of the median plate procurved and the posterior edge with a depression, and in having kidney-shaped spermathecae. However, *Phrynarachneyunhui* sp. nov. can be distinguished by the oval median plate (vs M-shaped in *P.mammillata*) and the broad anterior edge of the spermathecae (vs narrow in *P.mammillata*).

##### Description.

**Female** (Figs 15, 19D): total length 10.04, carapace 3.67 long, 4.44 wide, black. Eye sizes and interdistances: ALE 0.22, AME 0.19, PLE 0.25, PME 0.20; ALE–AME 0.16, AME–AME 0.27, PLE–PME 0.35, PME–PME 0.29. Chelicerae black, with two promarginal teeth and one retromarginal tooth; Gnathocoxae, labium black, labium 0.83 long, 0.75 wide. Sternum black. Legs black, femora I and II with dense, varying-sized tubercles; tibiae and metatarsi I, II with dense asymmetrical ventral spines (I, tibia 17, metatarsus 41; II, tibia 16, metatarsus 36). Leg measurements: I 13.01 (4.38, 4.63, 2.56, 1.44), II 12.74 (4.33, 4.58, 2.43, 1.40), III 6.78 (2.16, 2.42, 1.18, 1.02), IV 6.44 (2.35, 2.19, 1.07, 0.93). Leg formula: 1234. Opisthosoma grey, with dense, varying-sized, red-brown tubercles, each with a clavate seta.

Epigyne (Fig. 15). Sclerotized margins inconspicuous; median plate obvious, with a small posterior hood, anterior and posterior edges recurved, the ratio of width to length is 15:4; copulatory opening inconspicuous; spermathecae kidney-shaped, the ratio of posterior edge to anterior edge length is 1:1. Fertilization duct transverse.

##### Distribution.

Known only from the type locality.

#### ﻿ Phrynarachne zhengzhongi

Taxon classificationAnimaliaAraneaeThomisidae

Lin & S. Li sp. nov.

D921287B-2E97-568E-B1F0-B83E05EC2BBB

http://zoobank.org/77674036-F817-4088-A525-2DF0AB763CDC

Figs 16 , 20 , 21

##### Type material.

***Holotype***: ♀ (IZCAS-Ar41674), **China: *Yunnan***: Xishuangbanna, Jinghong City, Guanping Town, Shiwudui, 22.2310°N, 100.9172°E, 872 m elev., 27.IV.2018, Zhengzhong Huang leg. ***Paratype*** ♀ (IZCAS-Ar41675), same data as holotype.

##### Etymology.

The species is named after Mr Zhengzhong Huang, who collected the holotype and paratype; noun (name) in genitive case.

**Figure 16.**
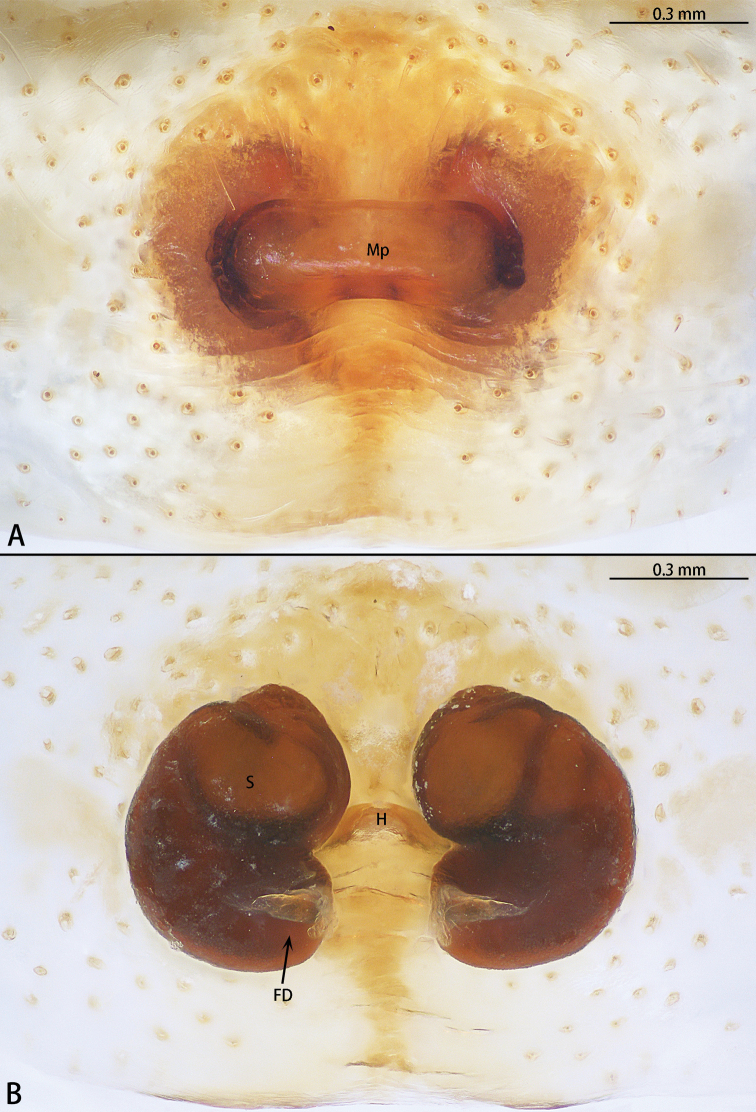
*Phrynarachnezhengzhongi* sp. nov., holotype female **A** epigyne, ventral **B** vulva, dorsal.

**Figure 17.**
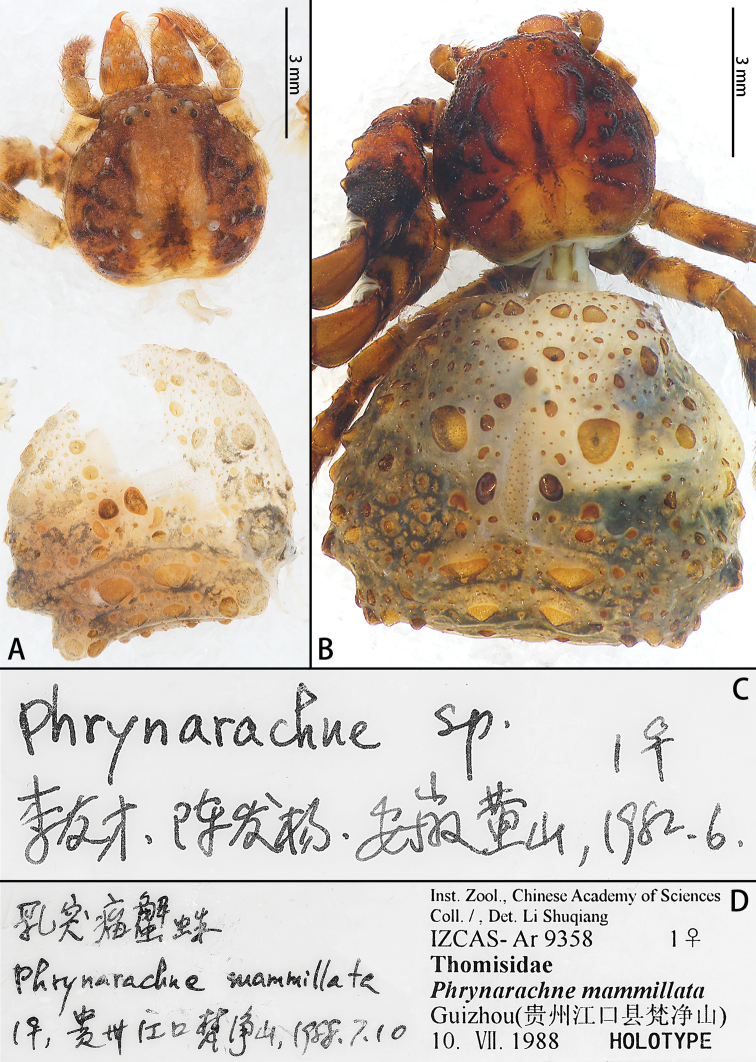
*Phrynarachne* spp., holotype females **A, C** habitus (**A**) and original labels (**C** handwriting by Daxiang Song) of *P.huangshanensis***B, D** habitus (**B**) and original label (**D** handwriting by Daxiang Song) of *P.mammillata*.

**Figure 18.**
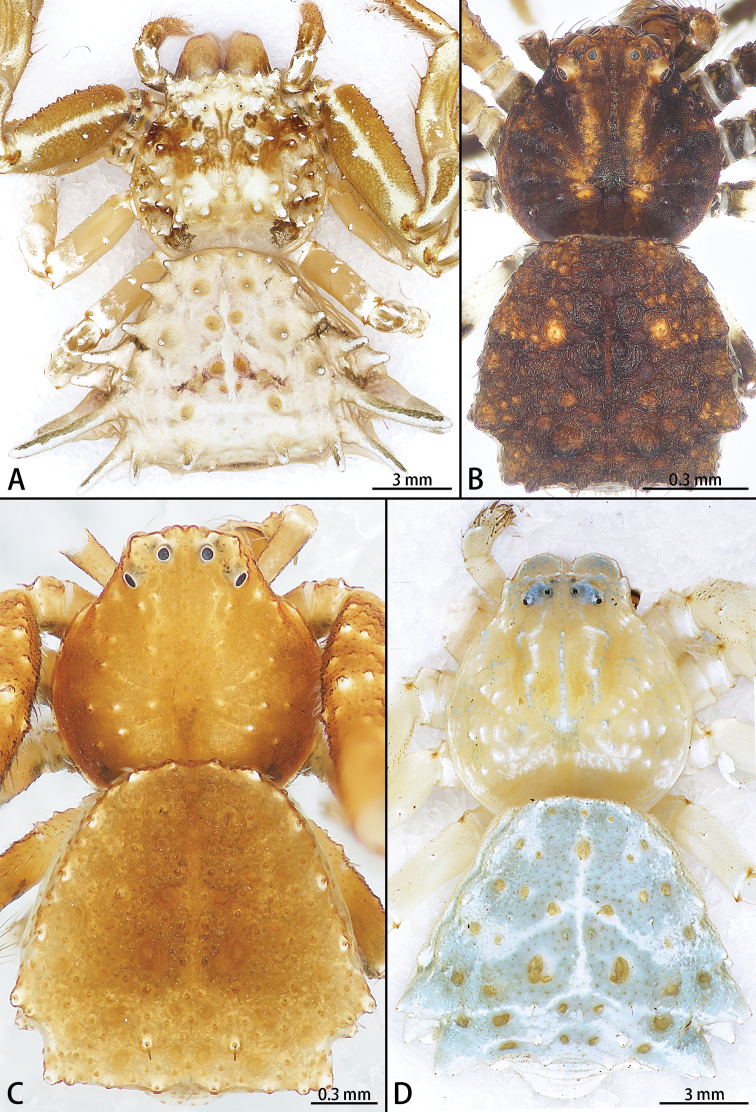
*Phrynarachne* spp., habitus dorsal **A***P.brevis*, female **B***P.huangshanensis*, male **C***P.dreepy* sp. nov., holotype male **D** same, paratype female.

**Figure 19.**
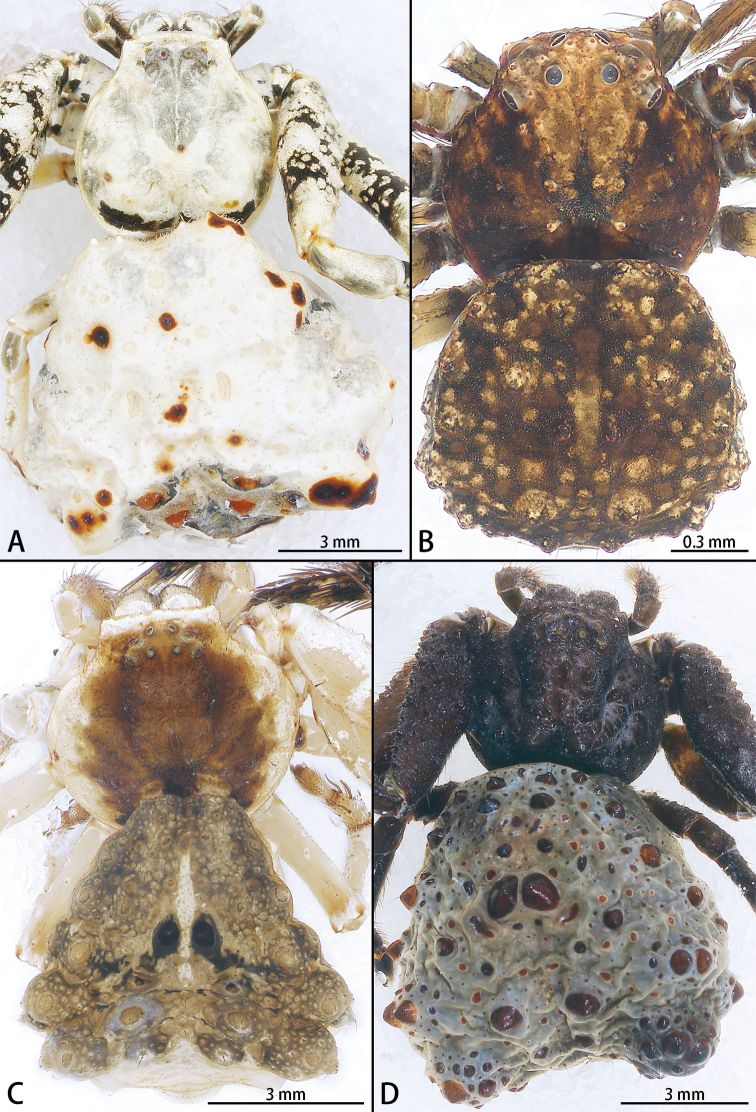
*Phrynarachne* spp., habitus dorsal **A***P.lancea*, female **B***P.mammillata*, male **C***P.xuxiake* sp. nov., holotype female **D***P.yunhui* sp. nov., holotype female.

**Figure 20.**
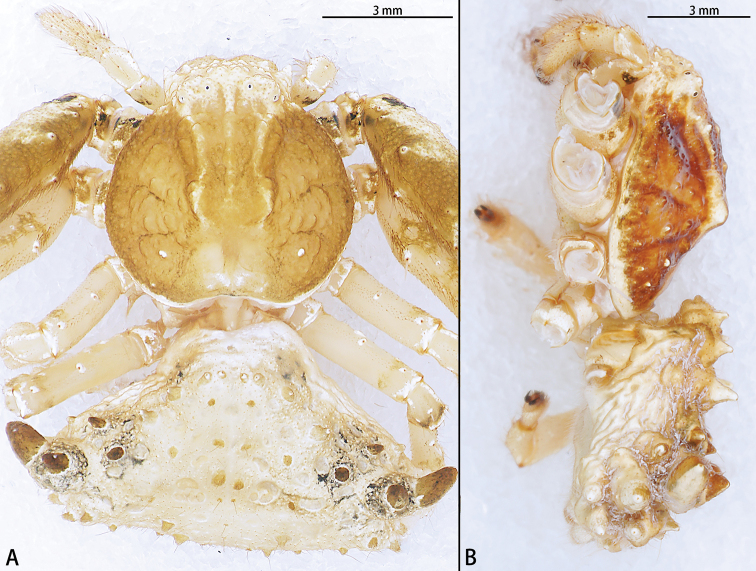
*Phrynarachnezhengzhongi* sp. nov., female holotype (**A**) and paratype (**B**) habitus **A** dorsal **B** lateral.

##### Diagnosis.

Females of *Phrynarachnezhengzhongi* sp. nov. are similiar to *P.brevis* by the shape of the spermathecae; the posterior edge of the spermathecae is as wide as the anterior edge. However, females of *P.zhengzhongi* sp. nov. can be distinguished by the triangular tubercles on the abdomen (vs long, slender apophysis in *P.brevis*), the epigyne with a hood, and the absence of sclerotized margins (vs hood absent, sclerotized margins present in *P.brevis*), and the straight anterior edge of the median plate (vs recurved in *P.brevis*).

##### Description.

**Female** (Figs 16, 20): total length 10.79, carapace 5.27 long, 5.53 wide, brown, with small projection, ocular tubercle white. Projection present between ALE and PLE. Eye sizes and interdistances: ALE 0.21, AME 0.17, PLE 0.18, PME 0.10; ALE–AME 0.35, AME–AME 0.66, PLE–PME 0.50, PME–PME 0.87. Chelicerae pale yellow, with two promarginal teeth and one retromarginal tooth; gnathocoxae, labium yellow, labium 1.19 long, 0.94 wide. Sternum yellow. Legs brown, femora I and II with dense, varying-sized tubercles; tibiae and metatarsi I, II with dense asymmetrical ventral spines (I, tibia 22, metatarsus 43; II, tibia 17, metatarsus 37). Leg measurements: I 14.43 (4.71, 5.91, 2.35, 1.46), II 14.78 (5.05, 6.31, 2.09, 1.33), III 7.76 (2.24, 3.48, 1.03, 1.01), IV 7.30 (2.44, 3.01, 0.92, 0.93). Leg formula: 2134. Opisthosoma dorsally light yellow, each side with 22 tubercles, each with some tubercles.

**Figure 21.**
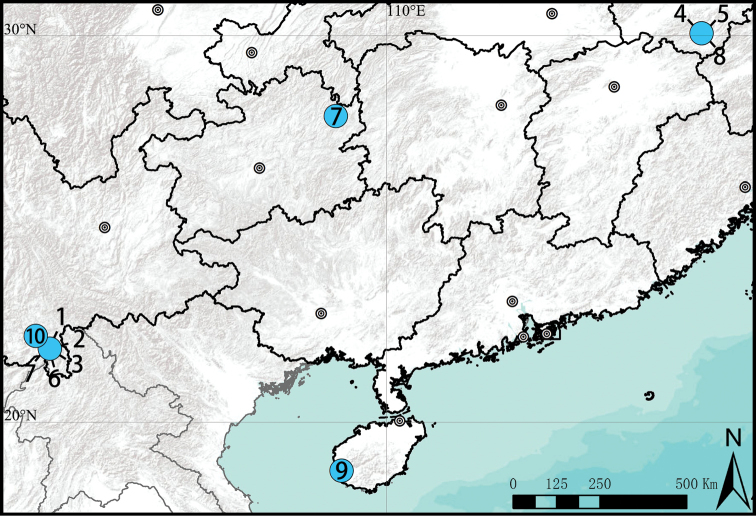
Distribution records of *Phrynarachne* species in China **1***P.brevis***2***P.ceylonica***3***P.dreepy* sp. nov. **4***P.huangshanensis***5***P.katoi***6***P.lancea***7***P.mammillata***8***P.xuxiake* sp. nov. **9***P.yunhui* sp. nov. **10***P.zhengzhongi* sp. nov.

Epigyne (Fig. 16). Sclerotized margins inconspicuous; median plate obvious, with a posterior hood, anterior and posterior edges almost straight, the ratio of width to length is 4:1; copulatory opening inconspicuous; spermathecae kidney-shaped, the ratio of posterior edge to anterior edge length is 1:1. Fertilization duct transverse.

##### Distribution.

Known only from the type locality.

## Supplementary Material

XML Treatment for Phrynarachne

XML Treatment for Phrynarachne brevis

XML Treatment for Phrynarachne ceylonica

XML Treatment for Phrynarachne dreepy

XML Treatment for Phrynarachne huangshanensis

XML Treatment for Phrynarachne katoi

XML Treatment for Phrynarachne lancea

XML Treatment for Phrynarachne mammillata

XML Treatment for Phrynarachne sinensis

XML Treatment for Phrynarachne xuxiake

XML Treatment for Phrynarachne yunhui

XML Treatment for Phrynarachne zhengzhongi
